# Associations of health literacy, dietary decisional balance, and dietary self-efficacy with dietary self-management behaviors in patients with type 2 diabetes mellitus: a cross-sectional study

**DOI:** 10.3389/fendo.2026.1942380

**Published:** 2026-10-28

**Authors:** Yan Wu, Yingwen Zhong, Qingyin Huang, Wenzhuan Chen, Yun Chen, Xu Liu

**Affiliations:** 1 School of Nursing, Guangzhou University of Chinese Medicine, Guangzhou, China; 2 The First Affiliated Hospital, Guangzhou University of Chinese Medicine, Guangzhou, China; 3 Department of Nursing, The Second Affiliated Hospital of Shantou University Medical College, Shantou, China; 4 Department of Nursing, Shenzhen Hospital (Futian) of Guangzhou University of Chinese Medicine, Shenzhen, Guangdong, China; 5 Department of Infectious Diseases, The Fifth Affiliated Hospital, Sun Yat-sen University, Zhuhai, China

**Keywords:** decision making, diabetes mellitus, type 2, diet, diabetic, health literacy, self-efficacy, self-management

## Abstract

**Background:**

Dietary self-management is essential for glycemic control in type 2 diabetes mellitus (T2DM), yet the factors associated with it and their interrelationships remain underexplored.

**Objective:**

To examine the associations of health literacy, dietary decisional balance, and dietary self-efficacy with dietary self-management behaviors in patients with T2DM.

**Methods:**

This was a cross-sectional study conducted in four endocrinology departments of four tertiary hospitals in Guangdong province from September 2022 to January 2023. A total of 387 inpatients diagnosed as T2DM were consecutively sampled. Health literacy was measured using the Health Literacy Scale (FCCHL). Dietary decisional balance was assessed using the Dietary Decisional Balance Scale, dietary self-efficacy using the diet-specific subscale of the Stanford Self-efficacy for Diabetes scale, and dietary self-management behaviors using the general diet domain of the Summary of Diabetes Self-Care Activities scale. The primary model was estimated using structural equation modeling in Mplus 7.0 with the robust maximum likelihood estimator (MLR). For inference on the indirect associations, the primary model was re-estimated using maximum likelihood (ML) with 5, 000 bootstrap draws, and bias-corrected 95% confidence intervals were obtained. A covariate-adjusted SEM was further estimated by including sociodemographic and clinical characteristics (age, sex, marital status, education, residence, occupation, income, smoking, disease duration, complications, and hospital).

**Results:**

Both the primary and dimension-specific SEMs showed good fit. A higher level of health literacy, dietary decisional balance, and dietary self-efficacy was associated with better dietary self-management behaviors. Health literacy showed a significant indirect association with dietary self-management behaviors through dietary self-efficacy (*β* = 0.077, 95% *CI*: 0.017 – 0.137, *p* = 0.012), but the indirect association through dietary decisional balance was not significant (*β* = 0.028, 95% *CI*: -0.006 – 0.062, *p* = 0.101). Dimension-specific analyses further showed that communicative health literacy was positively associated with dietary self-efficacy (*β* = 0.162, *p* = 0.034) and dietary self-management behaviors (*β* = 0.135, *p* = 0.043), whereas critical health literacy was positively associated with perceived benefits (*β* = 0.332, *p* < 0.01), and perceived barriers were negatively associated with dietary self-management behaviors (*β* = -0.166, *p* = 0.001).

**Conclusions and implications:**

Diabetes care should move beyond knowledge transmission by supporting patients’ communicative and critical health literacy, strengthening dietary self-efficacy, and identifying and addressing perceived dietary barriers to support dietary self-management behaviors in patients with T2DM.

## Highlights

- What is already known about the topic?
- Dietary self-management serve as the premise of diabetes self-management, yet adherence to dietary self-management behaviors remains suboptimal among patients with type 2 diabetes.
- A higher level of health literacy, better self-efficacy, and decisional balance were associated with better self-management behaviors among patients with multiple chronic diseases including type 2 diabetes.
- Evidence remains limited about the direct and indirect relationships among health literacy, dietary decisional balance, dietary self-efficacy, and dietary self-management behavior in patients with type 2 diabetes.

- What this paper adds
- This study provides preliminary support for a hypothesized model constructed based on the Framework of Health Literacy and Health Actions and trans-theoretical theory that described direct and indirect statistical associations between health literacy and dietary self-management behaviors through dietary decisional balance and dietary self-efficacy.
- A higher level of health literacy was associated with better dietary self-management behaviors both directly and indirectly through the mediation of dietary self-efficacy in patients with type 2 diabetes. Communicative health literacy was positively related to dietary self-efficacy and dietary self-management behaviors, whereas critical health literacy was positively associated with perceived benefits. Functional health literacy was not significantly associated with other study variables.
- A better dietary decisional balance was directly associated with better dietary self-management behaviors. Specifically, perceived barriers, rather than perceived benefits, were associated with poor dietary self-management behaviors.

## 1 Introduction

Type 2 diabetes mellitus (T2DM) has been identified as one of the most serious global public health problems. According to the latest global estimates of diabetes prevalence from the International Diabetes Federation (IDF) (1), more than 530 million adults worldwide have diabetes, accounting for 10.5% of the global adult population aged between 20–79 years old; more than 140 million adults with diabetes live in China, and the number continues to increase. An estimated 6.7 million people died from diabetes and its complications, accounting for 12.2% of global death in 2019 (1). People with diabetes were estimated to lose 4.4 years of life on average due to the disease (2, 3). Premature mortality, disability and work absenteeism associated with diabetes impose a substantial economic burden worldwide (4). The need to take effective measures to control diabetes, especially T2DM, which accounts for 90% of the diabetes, is clearer and more urgent than ever (5).

Diabetes self-management, including diabetes eating, physical activity, blood glucose monitoring, diabetes medication, and reducing the risks of complications, has been well-recognized as essential for controlling T2DM (6). Although diabetes self-management is multifaceted, dietary self-management serves as the premise of all facets because diet is directly associated with glycemic control. Dietary management is one of the safest and most modifiable aspects of diabetes care and may enhance the effectiveness of glucose-lowering therapy compared with medication alone (7, 8). Although international studies have suggested that diet and medication are among the most commonly followed self-management recommendations among patients with T2DM, adherence to dietary self-management behavior remains suboptimal regardless of duration of illness (9–13).

In recent years, various interventions have been developed to improve self-management, including self-management education programs, motivational interviewing, peer-support, and technology-based interventions (14–16). However, the implications for clinical practice remain inconclusive because the findings have been mixed, and evidence for improving dietary self-management specifically remains limited (14). This highlights the need to further explore factors associated with dietary self-management in patients with T2DM and how these factors relate to its adoption and maintenance.

Poor health literacy is considered as a potential barrier to optimal self-management in people with chronic conditions, including T2DM. Health literacy is defined as the degree to which individuals have the capacity to obtain, process, and understand basic health information and services needed to make appropriate health decisions (17). Mounting evidence suggests that people with T2DM and poor health literacy tend to be less likely to perform well in their disease management, such as glycemic control, and more likely to develop complications (18–20). However, some studies have found health literacy to be only weakly associated with self-management, including dietary self-management; this may be because most of these studies assessed only functional health literacy rather than other dimensions, including communicative and critical health literacy (19, 21). Potential indirect factors may also contribute to the observed association between health literacy, especially its functional dimension, and self-management (22–24).

In the Framework of Health Literacy and Health Actions developed by von Wagner et al. (25), the key mechanisms underlying the relationships of health literacy and self-management were further explored. Motivational determinants (i.e., traditional social cognition constructs result in a decision about whether to attempt to perform a health action), such as beliefs and attitudes, and volitional determinants (i.e., objective and subjective action control to translate intentions into actions), such as self-efficacy, were identified as serving as indirect pathways in the relationships between health literacy and health actions, including access and use of health care, patient–provider interactions, and the management of health and illness (e.g. diabetes self-management). This coincides with the assumption of trans-theoretical theory that the individual’s decisional balance (i.e., the cognitive process weighting pros (facilitators) and cons (barriers) of making a change) and self-efficacy (i.e., degree of confidence in one’s ability to engage in change) are two important variables associated with behavioral change across the stages (26).

Many studies have shown that a higher level of health literacy is associated with better self-management behaviors, including dietary self-management domain, or better glycemic control via self-efficacy among patients with T2DM (27–30). Decisional balance has been examined as a psychosocial predictor of behavior change in diverse populations with chronic diseases including patients receiving dialysis, patients with cancer, patients with hypertension (31) and patients with T2DM (32). However, the indirect role of decisional balance between health literacy and self-management has only been investigated in patients with cancer (33). No evidence was found to explore the relationships among health literacy, dietary decisional balance, dietary self-efficacy, and dietary self-management behavior in patients with T2DM.

This study aimed to explore the associations of health literacy, dietary decisional balance, and dietary self-efficacy with dietary self-management behavior in patients with T2DM. A hypothesized model was constructed according to aforementioned theories (i.e., Framework of Health Literacy and Health Actions and trans-theoretical theory) (**Figure 1**). We hypothesized that a higher level of health literacy was associated with better dietary self-management behavior both directly and indirectly through dietary decisional balance and dietary self-efficacy.

**Figure 1:**
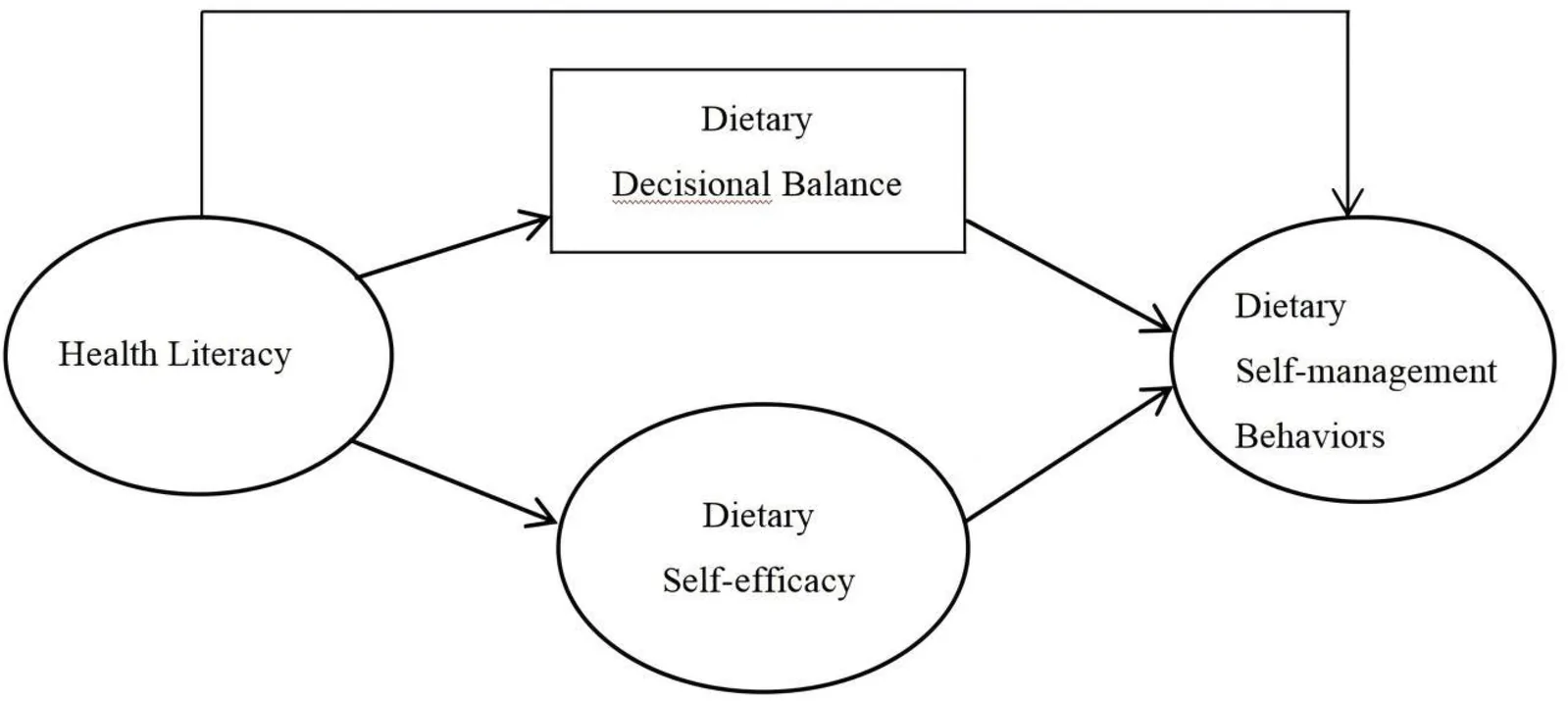
Hypothesized model.

## 2 Materials and methods

### 2.1 Design, setting, and participants

This was a cross-sectional study conducted in four endocrinology departments of four tertiary hospitals in Guangdong province from September 2022 to January 2023. Inpatients who were diagnosed with T2DM according to the “Chinese Guidelines for the Prevention and Treatment of Type 2 Diabetes Mellitus (2017 Edition)” were consecutively sampled. We excluded the patients suffering from serious physical diseases and the patients with/had been diagnosed with mental disorders due to their inability to cooperate with the research. The sample size was determined using two complementary approaches. First, following the widely recommended at least 5:1 ratio of sample size to free parameters for SEM, the hypothesized model with 46 free parameters required at least 230 participants to ensure stable parameter estimation (34). These 46 free parameters comprised the 3 observed variables of health literacy (functional, communicative, and critical), the 2 observed variables of dietary decisional balance (perceived pros and cons), the 3 observed variables of dietary self-efficacy, the 2 observed variables of dietary self-management behavior, as well as the factor loadings, structural paths, covariances, and residual variances. Second, an *a priori* power analysis was performed using the semPower R package (35). With the degrees of freedom of the model (df = 19), *α* = 0.05, power = 0.80, and RMSEA = 0.06 as the target effect size for close fit, the analysis indicated that 301 participants were required to detect model misspecification. All eligible patients were asked to provide oral informed consent and fill out a questionnaire anonymously. Of 443 questionnaires distributed, 387 (87.4%) valid questionnaires were returned and analyzed with SEM. Such a sample size (n=387) would be adequate to model estimation and can achieve an excellent power of 0.99 according to the *post hoc* power analysis with G*Power 3.1 (α=0.05, effect size=0.15). The study was approved by the Ethics Committee of School of Nursing, Guangzhou University of Chinese Medicine(Approval No. K-2022-042).

### 2.2 Variables and measurements

Patient characteristics including gender, age, marital status, education level, residence (urban or rural), employment status, monthly income, smoking history, disease duration, complications, and hospital) were surveyed with a self-developed questionnaire. Health literacy, decisional balance, self-efficacy, and dietary self-management behavior among diabetic patients were measured with the following instruments endorsed by their original developers.

#### 2.2.1 Health literacy

A 14-item Functional Communicative and Critical Health Literacy scale (FCCHL) was used to measure the HL, which assessed health literacy beyond the functional level (i.e., patients’ skill on reading the health information) and explored the barriers related to health literacy in self-care such as patients’ abilities on health information extraction, communication, critical analysis, and use (36). The FCCHL consists of three domains, namely functional (4 items), communicative (5 items), and critical (5 items) health literacy and was tested to have acceptable internal consistency (Cronbach’s α=0.84, 0.77, and 0.65, respectively) and construct validity when examining in diabetic patients (36). Items were rated on a Likert 4-point scale, ranging from 1 (never) to 4 (often). The scores of the functional health literacy were reversed to keep consistent with the other domains, where higher scores indicated higher health literacy. The total score ranges for the functional, communicative, and critical health literacy domains are 4–16, 5–20, and 5–20. The mean scores of the three domains were served as the indicators of health literacy, a latent variable in the hypothesized model. The Chinese version scale translated by Zhao et al. was tested to have satisfactory reliability and validity (37). In this study, the Cronbach’s α of the functional, communicative, and critical HL scores were 0.87, 0.87, and 0.90, respectively, which indicated acceptable internal consistency.

#### 2.2.2 Dietary decisional balance

The Dietary Decisional Balance Scale was used to assess the internal decision-making process (i.e., perceived pros and cons) of patients with T2DM in deciding whether or not to follow a diabetic diet (38). A total of 13 items in two domains, namely perceived pros (7 items) and the perceived cons (6 items) of diabetes diet compliance were included in the scale. At the beginning of the scale, specific definitions used for diabetes dietary are given to the patients: diabetes diet refers to arranging all your food and drinks for each meal and snack under the guidance of professionals (such as doctors, nurses, nutritionists). This includes (1) a reasonable control of the total daily energy to maintain an ideal body weight; (2) a control of the balance of daily carbohydrates, proteins, and fats; (3) eating three meals at regular intervals and quantities of three meals. This means eating the right kinds of food in the exact amounts at the right times of the day every day (39). Patients were asked to rate the importance of each pros and cons when they made a decision on the adherence to a diabetic diet from “not important” (score=1) to “very important” (score=5). The score of dietary decisional balance ranged from -4 to 4 was calculated by the mean item score of pro items minus the mean item score of con items and used as an observed variable. For example, if a patient rates all con items as “very important” (score 5) and all pro items as “not important” (score 1), the resulting score is 1 − 5 = −4. The higher the score, the more greatly the perceived benefits outweighed the disadvantages.

Our research group translated the scale to Chinese using standard forward- and back-translation procedures and cultural adaption (39). The Chinese version demonstrated good reliability and validity for assessing dietary decisional balance among patients with diabetes in the Chinese cultural context. The scale-level content validity index was 0.98, the item-level content validity indices ranged from 0.86 to 1.00, and Cronbach’s α coefficients for the total scale and subscales ranged from 0.730 to 0.899 (39). In this study, the Cronbach’s α of the pros and cons domains were 0.90 and 0.75.

#### 2.2.3 Dietary self-efficacy

The dietary self-efficacy was measured by the 3 items related to diet from the Stanford Self-efficacy for Diabetes (SED) scale developed by Lorig et al. (40). The full SED scale consists of 8 items assessing confidence across four domains: diet, exercise, blood glucose management, and disease control. The internal consistency reliability of the SED was 0.83 (41). The translated and adapted Chinese version of the scale was from Zhang et al. and the internal consistency (Cronbach’s α=0.890) and the validity of the scale were satisfactory according to their findings (42). The three-item diet subscale assesses confidence in following a diabetic eating plan and has been widely used as a separate domain of diabetes self-efficacy in previous studies (43). In this study, the 3 items related to diet with a Likert-5 scale (1= have no confidence at all, 5=have absolute confidence) were used as three indicators of the latent variable, patient dietary self-efficacy in the SEM. The Cronbach’s α of the dietary sub-domain (including 3 items) was 0.84 in this study.

#### 2.2.4 Dietary self-management behavior

The self-management behavior of patients on diet was measured with the two relevant items of the general diet domain from the widely used Summary of Diabetes Self-Care Activities (SDSCA) developed by Toobert (44). SDSCA is a brief self-report questionnaire of diabetes self-management. The instrument operationalized diabetes self-management by assessing the frequency of specific self-care behaviors, including diet, exercise, blood glucose testing, foot care, and smoking.The two-item diet subscale assesses the frequency of following a healthy eating plan and has been widely used as a separate domain of diabetes self-management in previous studies (45). Patients were asked to answer How many of the last seven days have you followed a diabetic eating plan? and On average, over the past month, how many days per week have you followed your eating plan? with a 0–7 answer scale, where a higher score indicates better dietary self-management behavior. Wan et al. (46) translated the scale and examined the psychometric properties of the scale. The Cronbach’s α of the general diet domain (0.89) and the construct validity from the exploratory factor analysis showed acceptable reliability and validity (46). The two items of the general diet domain served as two indicators of the dietary self-management behavior, a latent variable in the SEM (Cronbach’s α=0.87 in this study).

### 2.3 Data analysis

Sample size calculation was performed using R version 4.4.3 with the semPower package (35). Descriptive statistics for patient characteristics, health literacy, dietary decisional balance, dietary self-efficacy, and dietary self-management behaviors were conducted using SPSS Version 20.0. Covariates were selected based on empirical evidence from systematic reviews and meta-analyses (47–52) The intraclass correlation coefficient (ICC) was calculated to estimate the proportion of variance in the dependent variable (i.e., dietary self-management behaviors) attributable to hospital-level clustering. The value of ICC (0.05) indicated that only 5% of the total variation in dietary self-management behavior can be accounted for by which department/hospital each participant was in and thus a fixed effect model was used. Then, the primary model was estimated using structural equation modeling in Mplus Version 7 with the robust maximum likelihood estimator (MLR) for model fit and parameter estimation. For inference on indirect associations, the primary model was re-tested using maximum likelihood (ML) with 5, 000 bootstrap draws, and bias-corrected 95% confidence intervals were reported. A covariate-adjusted primary model was further estimated by including age, sex, marital status, education, residence, occupation, income, smoking, disease duration, complications, and hospital. To examine the robustness of the findings, we further conducted dimension-specific analyses based on the following consideration. First, given the low factor loading of the functional health literacy domain on the overall health literacy latent variable, we modeled functional, communicative, and critical health literacy as three separate observed variables instead of a single latent construct. Second, to further examine the potentially distinct role of two components of dietary decisional balance, dietary decisional balance was split into its pros and cons subscales. The dimension-specific model was analyzed using the same strategy as the primary model. Model fit was evaluated according to the standardized root mean square residual (SRMR; < 0.08); comparative fit index (CFI; ≥ 0.90), Tucker-Lewis index (TLI; ≥ 0.90); and root mean square error of approximation (RMSEA; < 0.08) (53). There was no more than 10% missingness in the items used in the study. Full-information maximum likelihood estimation was used to cope with the missing outcome data (53). Normality was assessed using the Shapiro–Wilk test and skewness/kurtosis. As data deviated from normality, the MLR estimator was used to handle non-normality.

## 3 Results

### 3.1 Patient characteristics

The patient characteristics were presented in **Table 1**. About half of the patients were female. Most of the patients were over the age of 45, had a spouse, completed junior/senior high school education, and were living in urban areas. More than 60% of the patients had a job and more than half of the patients had at least 3000 RMB income per month. About 30% of the patients had a history of smoking. About half of the patients had a long course of T2DM (i.e., ≥ 5 years) and multiple complications related to T2DM. Participants were recruited from four tertiary hospitals: 147 (38.0%) from hospital 1, 59 (15.2%) from hospital 2, 76 (19.6%) from hospital 3, and 105 (27.1%) from hospital 4.

**Table 1:** Patient characteristics (N = 387)^a^.

Characteristics	n (%)	Characteristics	n (%)
Gender (female)	163 (43.4)	Monthly income ^c^	
Age ^b^		No fixed income	73 (19.4)
18 – 44	60 (15.7)	< 3000 RMB	109 (29.0)
45 – 64	187 (49.1)	≥ 3000, < 4999 RMB	96 (25.5)
65 – 89	134 (35.2)	≥ 5000 RMB	98 (26.1)
Marital status (have a spouse)	294 (76.6)	Length of disease	
Education level		< 3 months	67 (17.4)
No schooling/primary school	87 (22.8)	≥ 3 months, < 3 years	75 (19.5)
Junior/senior high school	205 (53.6)	≥ 3 years, < 5 years	36 (9.4)
College and higher	90 (23.6)	≥ 5 years	206 (53.7)
Place of residence		Complications related to T2DM ^d^ (Yes)	188 (48.6)
Urban areas	272 (73.1)	Hospital	
Rural areas	100 (26.9)	Hospital 1	147 (38.0)
Smoking history (Yes)	113 (30.1)	Hospital 2	59 (15.2)
Job status		Hospital 3	76 (19.6)
Employed	228 (60.2)	Hospital 4	105 (27.1)
Unemployed/Retired	151 (39.8)		

^a^ Sample size for different characteristics varied due to missing data. ^b^ The mean age was 57.5 ± 14.1 years old. ^c^ 1 RMB = 0.15 US dollar (September 2, 2021). ^d^ Complications related to T2DM included diabetic nephropathy, diabetic retinopathy, diabetic foot, diabetic neuropathy, and diabetic lower limb vascular disease.

### 3.2 Health literacy, dietary decisional balance, dietary self-efficacy, and dietary self-management behavior

In general, the patients with T2DM had moderate levels of functional, communicative, and critical health literacy (**Table 2**). When the patients deciding whether or not to follow a diabetic diet, their perceived benefits on following a diabetic diet slightly overweighted the barriers. The self-efficacy of the patients with T2DM on insisting on a diabetic diet was also moderate. As for the dietary self-management behaviors of the patients with T2DM, they followed a diabetic eating plan for about 5 days per week on average. However, 30% – 40% of the patients followed a diabetic diet ≤ 4 days per week. The Shapiro–Wilk test results indicated that most variables deviated from normality (*p* < 0.05). Therefore, the MLR estimator was used to handle non-normality. The factor loading value of the indicators showed that the latent variables were well measured except for the functional domain of health literacy (**Table 3**).

**Table 2:** Descriptive statistics of study variables (N = 387)^a^.

Variable	Min	Max	Mean ± SD
Health literacy (HL)			
Functional HL	1	4	2.73 ± 0.87
Communicative HL	1	4	2.86 ± 0.80
Critical HL	1	4	2.71 ± 0.93
Total FCCHL score	14	56	38.86 ± 8.62
Dietary decisional balance			
Perceived benefits(pro)	7	35	28.56 ± 4.80
Perceived barriers(con)	6	30	16.95 ± 4.53
Decisional balance score	-2.31	3.83	1.26 ± 1.11
Dietary self-efficacy(SED)			
Item 1: Eat meals regularly	1	5	3.49 ± 1.20
Item 2: Follow diet when sharing food	1	5	3.54 ± 1.10
Item 3: Choose appropriate foods when hungry	1	5	3.47 ± 1.10
Total SED diet score	3	15	10.49 ± 2.93
Dietary self-management			
Item 1: Followed a healthful eating plan	0	7	5.05 ± 2.38
Item 2: Followed eating plan per week	0	7	4.73 ± 2.48

^a^ HL, Health Literacy scale; SED, Self-efficacy for Diabetes scale; SDSCA, Summary of Diabetes Self-Care Activities scale. Health literacy dimensions are domain means; perceived benefits(pro) and barriers(con) are domain sums; decisional balance is pros mean minus cons mean; individual items are presented as item-level scores.

**Table 3:** Descriptions and factor loadings of study variables.

Latent variables (L) and observed variables (O)	Mean ± SD	Range	Factor loading	SE	95% *CI*
Health literacy (HL; L)					
Functional HL (O)	2.73 ± 0.87	1 – 4	0.13	0.065	0.000 – 0.255
Communicative HL (O)	2.86 ± 0.80	1 – 4	0.82**	0.144	0.543 – 1.106
Critical HL (O)	2.71 ± 0.93	1 – 4	0.79**	0.112	0.562 – 1.002
Dietary decisional balance (O)	1.26 ± 1.11	-4 – 4	—	—	—
Dietary self-efficacy (L)					
Item 1: Eat meals regularly (O)	3.49 ± 1.20	1 – 5	0.77**	0.034	0.703 – 0.838
Item 2: Follow your diet when have to prepare or share food with non-diabetic people (O)	3.54 ± 1.10	1 – 5	0.91**	0.028	0.852 – 0.963
Item 3: Choose the appropriate foods to eat when hungry (O)	3.47 ± 1.10	1 – 5	0.73**	0.036	0.661 – 0.803
Dietary self-management behavior (L)					
Item 1: Followed a healthful eating plan (O) ^a^	5.05 ± 2.38	0 – 7	0.88**	0.040	0.799 – 0.955
Item 2: Followed the eating plan per week on average over the past month (O) ^b^	4.73 ± 2.48	0 – 7	0.88**	0.041	0.800 – 0.962

^a^ 34.5% of the patients followed a healthful eating plan ≤ 4 days per week; ^b^39.1% followed the eating plan ≤ 4 days per week on average over the past month. ^**^p < 0.01.

### 3.3 Model performance and parameter estimates

#### 3.3.1 Primary model

The primary model fit well with the data [CFI = 0.972, TLI = 0.955, SRMR = 0.035, and RMSEA = 0.056 (95% *CI*: 0.035 – 0.077)]. We also tested an adjusted model with all the patients’ characteristics controlled and found that the direct and indirect estimates among the study variables were comparable to the final model unadjusted (**Figure 2**). The model explained 27.3% of the variance in dietary self-management behavior.

**Figure 2:**
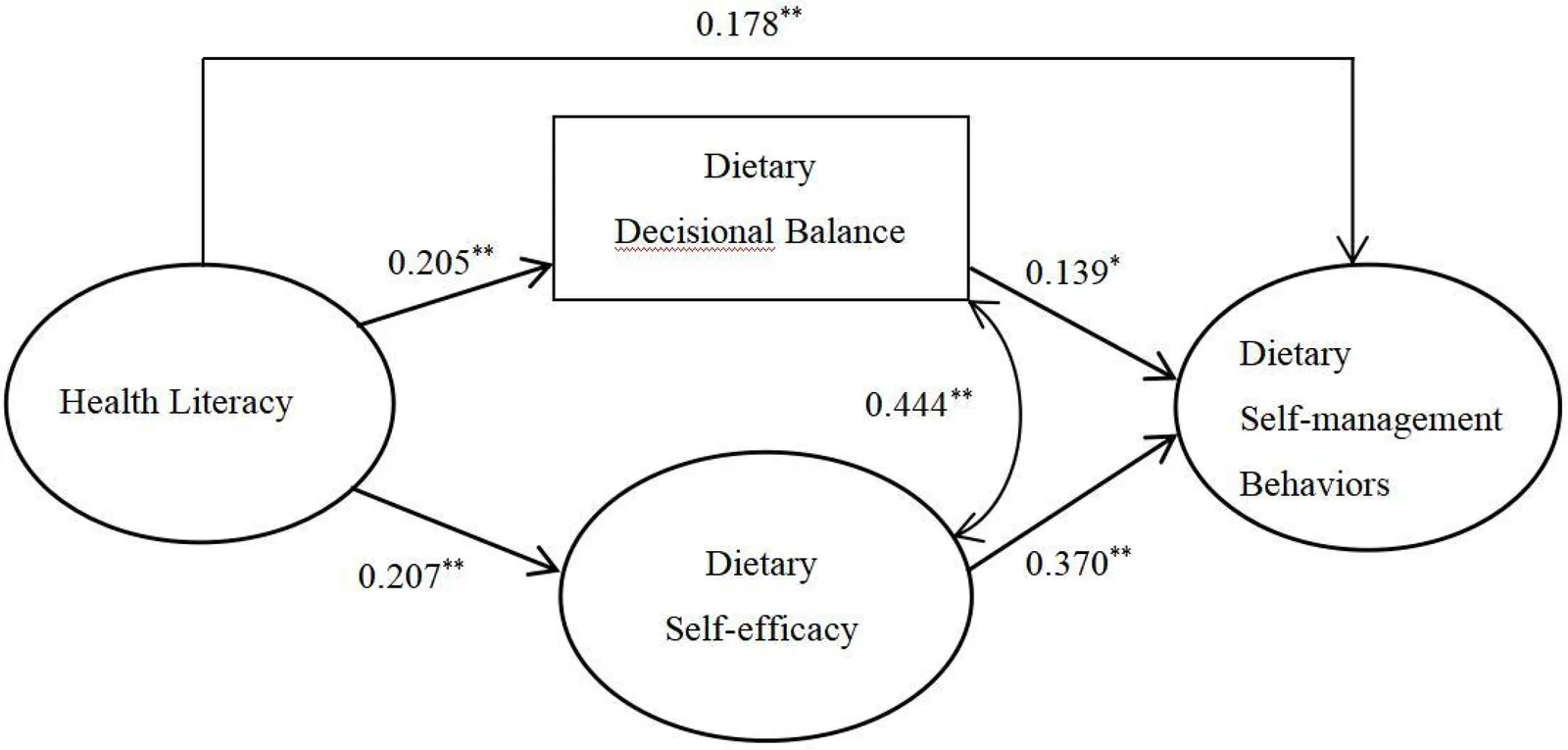
Final model with standardized coefficients. Model fit for the primary model: *χ²* = 49.914, *df* = 22, *p* = 0.0006; CFI = 0.972, TLI = 0.955, RMSEA = 0.056 (95% *CI*: 0.035–0.077), SRMR = 0.035. **p < 0.01, *p < 0.05.

A higher level of health literacy, dietary decisional balance, and dietary self-efficacy were directly associated with better dietary self-management behaviors. In addition, a higher level of health literacy was indirectly associated with better dietary self-management behaviors through dietary self-efficacy (*β* = 0.077, 95% *CI*: 0.017 – 0.137, *p* = 0.012). Although there were significant direct associations between health literacy and dietary decisional balance, the indirect path from health literacy to dietary self-management behavior via dietary decisional balance was not significant (*β* = 0.028, 95% *CI*: -0.006 – 0.062, *p* = 0.101).

#### 3.3.2 Dimension-specific model

Without any covariates, the dimension-specific model showed excellent fit [CFI = 0.983, TLI = 0.962, RMSEA = 0.046 (95% *CI*: 0.020 – 0.070), and SRMR = 0.024]. Consistent with the main findings, communicative health literacy was positively associated with dietary self-efficacy (*β* = 0.162, *p* = 0.034) and dietary self-management behavior (*β* = 0.135, *p* = 0.043), whereas critical health literacy was positively associated with perceived benefits (*β* = 0.332, *p* < 0.01). Functional health literacy showed no significant associations with the study variables. Among the components of dietary decisional balance, perceived benefits were not significantly associated with dietary self-management behaviors (*β* = 0.012, *p* = 0.834), whereas perceived barriers were negatively associated with dietary self-management behavior (*β* = -0.166, *p* = 0.001). Dietary self-efficacy remained positively associated with dietary self-management behavior (*β* = 0.383, *p* < 0.001). The indirect path from communicative health literacy to dietary self-management behavior via self-efficacy remained significant (*β* = 0.062, *p* = 0.049, 95% *CI*: 0.000 – 0.124) (**Figure 3**). The model explained 27.8% of the variance in dietary self-management behavior.

**Figure 3:**
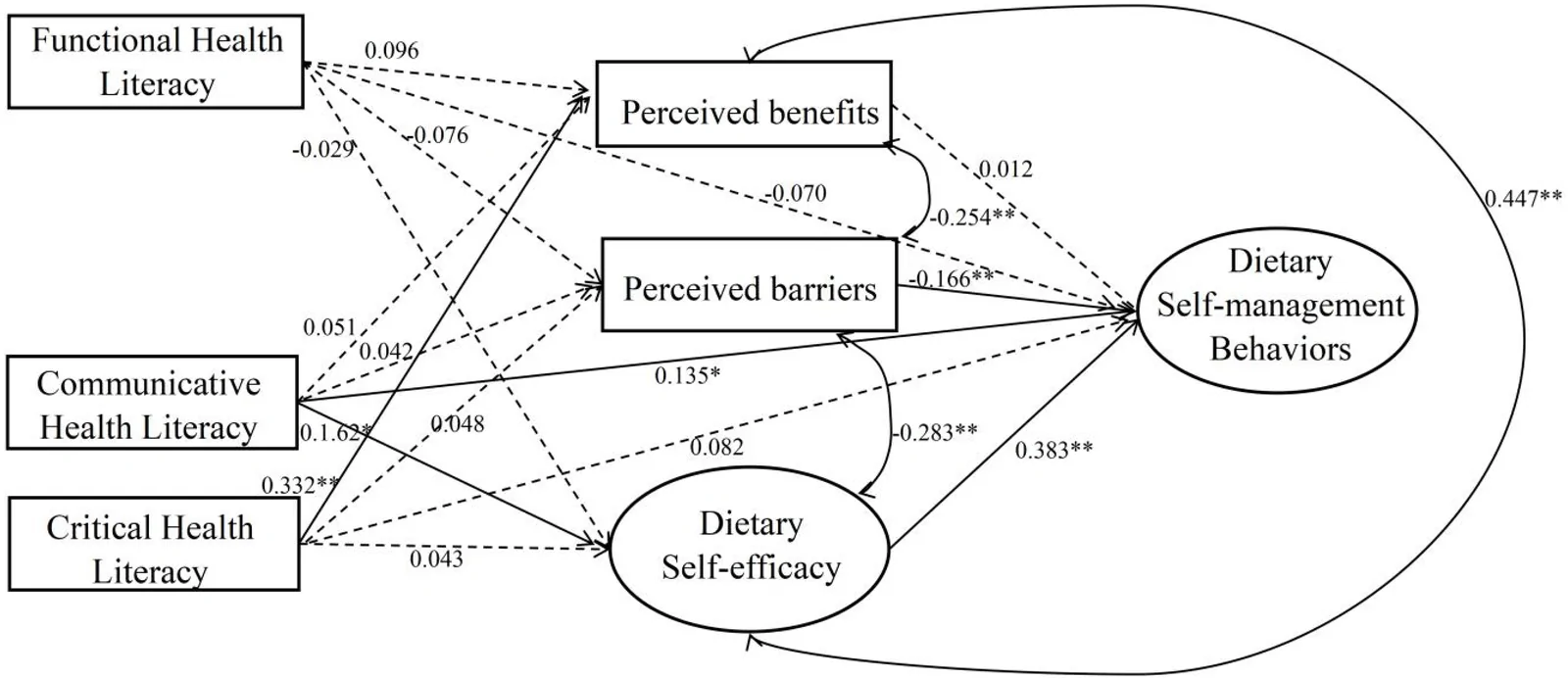
Dimension-specific analyses model with standardized coefficients. Model fit: *χ²* = 34.419, *df* = 19, *p* = 0.0164; CFI = 0.983, TLI = 0.962, RMSEA = 0.046 (95% *CI*: 0.020–0.070), SRMR = 0.024.**p < 0.01, *p < 0.05.

To further control for potential confounding, we then included all patient characteristics (gender, age, marital status, education, residence, occupation, monthly income, smoking history, disease duration, complications, and four hospitals) as covariates. This model showed acceptable fit [CFI = 0.915, TLI = 0.878, RMSEA = 0.057 (95% *CI*: 0.045 – 0.070), and SRMR = 0.060]. After adjustment, the overall paths of associations remained consistent with that observed in the dimension-specific model. Hospital fixed effects were not significant, and covariate effects are presented in **Table 4**.

**Table 4:** Associations between covariates and endogenous variables in the dimension-specific model.

Covariates	Dietary self-efficacy	Perceived benefits	Perceived barriers	Dietary self-management behaviors
Age	0.160*	-0.152*	-0.141*	0.031
Married	-0.067	0.045	0.059	-0.122*
Residence	-0.019	0.015	0.012	0.048
Job	-0.026	0.031	0.016	0.082
Income	-0.059	-0.049	0.001	0.111
Smoking	-0.136*	-0.071	-0.061	-0.004
Disease duration	0.015	0.023	0.041	-0.012
Complications	0.129*	-0.055	-0.116	-0.150**
Hospital 2 (vs. Hospital 1)	-0.002	0.042	0.020	0.015
Hospital 3 (vs. Hospital 1)	-0.071	0.066	-0.018	0.028
Hospital 4 (vs. Hospital 1)	-0.075	-0.022	0.033	0.044

^a^ All covariates were entered simultaneously in the covariate-adjusted dimension-specific model. Hospital 2, Hospital 3, and Hospital 4 are dummy variables with Hospital 1 as the reference category. **p < 0.01, *p < 0.05.

## 4 Discussion

This cross-sectional study aimed to examine the associations of health literacy, dietary decisional balance, and dietary self-efficacy with dietary self-management behaviors in patients with T2DM. Both SEM models showed good model fit. The primary SEM showed that health literacy, was positively related to dietary decisional balance, dietary self-efficacy and dietary self-management behaviors, and also showed a significant statistical indirect path to dietary self-management behaviors through dietary self-efficacy, whereas the indirect path through dietary decisional balance was not significant. Further dimension-specific analyses revealed distinct patterns of associations of three dimensions of health literacy with other study variables. Communicative health literacy was positively related to dietary self-efficacy and dietary self-management behaviors, whereas critical health literacy was positively associated with perceived benefits; perceived barriers, rather than perceived benefits, were negatively associated with dietary self-management behaviors. These findings clarified the distinct roles of the three health literacy dimensions and helped explain the non-significant indirect path through dietary decisional balance.

The patients with T2DM in this study followed a diabetic diet for about 5 days per week on average, which was comparable to findings reported in Italy, Malaysia, and the United States (10, 54, 55). However, there were still 30% to 40% of the patients followed the diabetic diet on 4 days per week or fewer, indicating a continuing need for practical dietary management support. In addition, hospital fixed effects were not significant in the adjusted models, suggesting that the observed associations were not substantially influenced by between-hospital differences.

Health literacy showed a significant indirect statistical path to dietary self-management behaviors via dietary self-efficacy. Specifically, communicative health literacy showed the clearest relationship with dietary self-management. Communicative health literacy reflects the ability to extract and derive meaning from health information obtained through different forms of communication and applying new information to changing circumstances. Our result showed it was associated both with dietary self-efficacy and with dietary self-management behaviors. This was consistent with previous studies reporting associations of communicative health literacy with self-efficacy and self-management (29, 52). Dimension-specific analyses further showed that communicative health literacy played an important role in this mediating pathway (28, 36). The present findings therefore suggest that diabetes education should extend beyond the transmission of dietary knowledge. In clinical and community programs, greater attention could be given to whether patients can discuss dietary information with health professionals, interpret recommendations in relation to their own circumstances when making daily food choices. Such an approach may be more informative than evaluating knowledge acquisition alone.

In contrast, functional health literacy, which mainly reflects basic reading and comprehension skills, was not significantly associated with dietary decisional balance, dietary self-efficacy, or dietary self-management behaviors. The covariate analysis showed that older age was associated with higher dietary self-efficacy, lower perceived benefits and perceived barriers, but was not significantly associated with dietary self-management behaviors, possibly reflecting more established dietary routines. Together, these findings suggest that limitations in basic functional health literacy may not necessarily correspond to poorer confidence or dietary self-management. This may be particularly relevant for older adults with age-related declines in reading or information-processing abilities. Rather than viewing such limitations as an inevitable barrier to self-management, diabetes care should adapt support strategies to age-related limitations in health literacy and functional abilities through accessible information, simplified communication, and continuous support (56).

Critical health literacy showed a different pattern of association, with a positive relationship with perceived benefits but no direct relationship with dietary self-management behaviors. However, the dimension-specific model provide a more differentiated view of dietary decisional balance. Perceived benefits were not directly associated with dietary self-management behaviors, whereas perceived barriers appeared to be more directly associated with poorer dietary self-management behaviors. Critical health literacy involves evaluating and judging health information and using it to make informed decisions. Patients with stronger critical health literacy may therefore be better able to recognize the potential benefits of dietary management. In addition to helping patients translate their information appraisal and judgment abilities and positive perceptions into feasible dietary decisions, diabetes care should strengthen the assessment of dietary barriers and provide targeted support. Previous studies have shown that dietary adherence in patients with T2DM can be constrained by family and social contexts, financial costs, work schedules, and established dietary habits (50, 57). Consistently, our covariate analysis suggested that marital status, complications, and smoking history were also related to self-efficacy or dietary self-management, indicating that family context, lifestyle habits, and disease burden may contribute to barriers to dietary self-management. Therefore, rather than simply informing patients what they should eat, healthcare professionals should identify the reasons why recommended dietary behaviors are difficult to implement (58).

In both the primary and dimension-specific models, dietary self-efficacy showed an indirect role between health literacy and dietary self-management behaviors, which was consistent with the findings on the relationships among health literacy, general self-efficacy, and diabetes self-management (27–30). According to Bandura et al. (61), self-efficacy perceptions are associated with what individuals do with the knowledge and skills they have. Patients with higher health literacy can have more confidence in their capabilities to behave well in self-management and more tend to achieve them actually (30). The dietary self-efficacy, as suggested by the findings, could be an essential target of interventions to enhance dietary self-management on the one hand, and on the other hand, to promote health equity related to health literacy needed for diabetes management (30). In addition to improving health literacy as indicated by the findings of this study and previous studies (5, 59), other interventions and strategies such as self-management education with family support, mindful eating intervention, and dietary calorie calculation and reporting may also be helpful for enhancing dietary self-efficacy, and thereby regulate dietary self-management performance in patients with T2DM (60).

### 4.1 Limitations

There are several limitations to our study. First, causal mediation cannot be inferred from this cross-sectional design. The indirect paths are statistical associations, not causal mediation, and require longitudinal or experimental verification. Second, although the model fit the data well, only 27.3% and 27.8% of the variance in dietary self-management behaviors were explained by the primary and dimension-specific models. Further research is needed to explore factors associated with dietary self-management behaviors in patients with T2DM. Third, the dietary self-management behavior was only measured with a subjective measurement tool. Further studies are expected to add some objective indicators and verify the findings. Fourth, the participants in this study were generally well-educated, living in urban areas, and well-paid. Implications from this study may not be generalized to patients who are less educated, living in rural areas, and poorly paid.

### 4.2 Practice implications

Health literacy may be a relevant target for interventions aiming to improve dietary self-management. Compared with functional health literacy that addresses the skills of reading health information, more efforts should be taken to improve patients’ communicative and critical health literacy (i.e., the skills of extracting, obtaining, analyzing, and using health information), which were more strongly associated with dietary self-efficacy and self-management. Other strategies, such as assessing perceived dietary barriers, providing individualized support according to patients’ practical difficulties, and strengthening self-efficacy, may also support better dietary self-management performance in patients with T2DM.

## 5 Conclusions

This study provides preliminary support for a hypothesized model constructed based on the Framework of Health Literacy and Health Actions and trans-theoretical theory that described statistical associations between the health literacy and dietary self-management behaviors in patients with type 2 diabetes. Our findings suggest that diabetes care should move beyond knowledge transmission by supporting patients’ communicative and critical health literacy, strengthening dietary self-efficacy, and identifying and addressing perceived dietary barriers to support dietary self-management behaviors in patients with T2DM.

## Data Availability

The raw data supporting the conclusions of this article will be made available by the authors, without undue reservation.
